# Experience of 2003 SARS has a negative psychological impact on healthcare workers in the COVID-19 pandemic: a cross-sectional study

**DOI:** 10.1590/1516-3180.2020.0516.R1.10122020

**Published:** 2021-02-01

**Authors:** Chun-Hsien Chen, Pei-Hsuan Yang, Fang-Li Kuo, I-Jeng Yeh, Che-Yu Su

**Affiliations:** I MD, MSc. Attending Physician, Department of Emergency Medicine, Kaohsiung Medical University Hospital, Kaohsiung Medical University, Kaohsiung, Taiwan.; II PhD. Registered Nurse, Department of Nursing, Kaohsiung Medical University Hospital, Kaohsiung Medical University, Kaohsiung, Taiwan. Assistant Professor, School of Nursing, Fooyin University, Kaohsiung, Taiwan.; III MSc. Registered Nurse, Department of Nursing, Kaohsiung Medical University Hospital, Kaohsiung Medical University, Kaohsiung, Taiwan.; IV MD, MSc. Attending Physician, Department of Emergency Medicine, Kaohsiung Medical University Hospital, Kaohsiung Medical University, Kaohsiung, Taiwan. Assistant Professor, School of Medicine, College of Medicine, Kaohsiung Medical University, Kaohsiung, Taiwan.; V MD. Attending Physician, Department of Emergency Medicine, Kaohsiung Medical University Hospital, Kaohsiung Medical University, Kaohsiung, Taiwan. Master’s Student, Graduate school, Department of Public Health, College of Public Health Science, Kaohsiung Medical University, Kaohsiung, Taiwan.

**Keywords:** COVID-19 [supplementary concept], Severe acute respiratory syndrome, Occupational stress, Health personnel, Pandemics, Psychometric evaluation, Highly infectious disease, Total stress scores, Subscale.

## Abstract

**BACKGROUND:**

The COVID-19 pandemic has instilled fear and stress among healthcare workers.

**OBJECTIVES:**

The aim of this study was to assess work stress and associated factors among healthcare workers during the COVID-19 outbreak and to evaluate whether prior experience of treating severe acute respiratory syndrome (SARS) had a positive or negative influence on healthcare workers’ stress levels during the COVID-19 pandemic.

**DESIGN AND SETTING:**

Cross-sectional survey in a tertiary hospital in Kaohsiung City, in southern Taiwan.

**METHODS:**

The survey was conducted using an online self-administered questionnaire to measure the stress levels among healthcare workers from March 20 to April 20, 2020. The stress scales were divided into four subscales: worry of social isolation; discomfort caused by the protective equipment; difficulties and anxiety regarding infection control; and workload of caring for patients.

**RESULTS:**

The total stress scores were significantly higher among healthcare workers who were aged 41 or above, female, married, parents and nurses. Those with experience of treating SARS reported having significantly higher stress scores on the subscale measuring the discomfort caused by protective equipment and the workload of caring for patients. During the COVID-19 outbreak, frontline healthcare workers with experience of treating SARS indicated having higher stress levels regarding the workload of caring for patients than did non-frontline healthcare workers with no experience of treating SARS.

**CONCLUSIONS:**

Work experience from dealing with the 2003 SARS virus may have had a negative psychological impact on healthcare workers amidst the COVID-19 outbreak.

## INTRODUCTION

Infectious disease outbreaks are always important issues that need to be to learned from, tackled and prevented. The coronavirus disease 2019 (COVID-19), a novel virus causing pneumonia that was first reported in Wuhan, China, is spreading locally and internationally. According to a report from the World Health Organization (WHO),^1^ over three million people around the world were confirmed to have COVID-19 between December 2019 and April 30, 2020, and approximately 217,000 of them died, which resulted in a mortality rate of approximately 7%.

Symptoms of COVID-19 appear within about 2 to 14 days after infection with the virus, and the disease progresses rapidly from an asymptomatic state or mild symptoms to severe symptoms. COVID-19 is highly transmissible between humans, is associated with high morbidity rates, and may potentially lead to fatality. This instills fear not only among the public but also among healthcare workers. Moreover, healthcare workers involved in diagnosis, treatment and care of patients with COVID-19 are at greater risk of contracting the disease. Therefore, they may have a higher possibility of developing psychological distress and other mental health symptoms.

COVID-19 is similar to the severe acute respiratory syndrome (SARS) that first infected humans in the Guangdong province of China in November 2002 and quickly spread throughout several parts of the world from 2002 to 2003. The SARS outbreak affected 26 countries and resulted in more than 8,000 cases in 2003.^2^

Taiwan was unable to remain free from SARS and the first case of this disease appeared on March 10, 2003. It then spread to multiple regions of Taiwan. Between March and June 2003, there were 346 cases of SARS in Taiwan.^3^ This outbreak in 2003 not only caused extraordinary public health concerns but also led to tremendous psychological distress, particularly among healthcare workers. These workers suffered from acute stress disorders;^4^ feared contagion and infection of their families, friends and colleagues with the SARS virus; and felt stigmatized and rejected in their neighborhoods because of their hospital work.^4^ Furthermore, these healthcare workers reported feeling reluctance to work or had considered resigning.^4^^,^^5^ In addition, healthcare workers who cared for SARS patients but did not become infected continued to experience substantial psychological distress, even one to two years after the outbreak.^6^

## OBJECTIVE

We hypothesized that healthcare workers who had had care experience relating to SARS in 2003 might be more stressed during the COVID-19 outbreak in Taiwan. The goal of this study was to better understand what factors contributed to the levels of stress experienced by healthcare workers, what aspects of their work might put pressure on them, and whether their prior SARS epidemic experience had enabled or inhibited their work with regard to dealing with the pandemic. With the above information, hospital administrators may better understand how to take action to provide psychological support measures or interventions to reduce the burden on healthcare workers in future infectious pandemics.

## METHODS

### Study design and study population

A cross-sectional survey was conducted in a tertiary-level hospital in Kaohsiung City, in southern Taiwan, from March 20 to April 20, 2020, amidst the COVID-19 pandemic in Taiwan. The survey was conducted using a web-based questionnaire and excluded new recruits, outsourced workers, research assistants and other non-regular hospital employees. Details of the survey website were provided to the survey participants through their mailboxes and the researchers compiled the responses for analysis. For this study, participants were recruited from the three main categories of hospital staff who presented the possibility of close contact with the suspected COVID-19 patients or with specimens that had been obtained for testing, namely doctors, nurses and medical technicians. The medical technicians mentioned here were those who assisted in making medical diagnoses by performing tests that had been requested by physicians and hospitals, such as medical and radiological technologists.

The questionnaire contained the following two sections. First, there were items asking about the participants’ demographic characteristics, including age, gender, marital status, being a parent, occupation, educational level, years of work experience, experience of caring for patients with SARS and experience of caring for suspected or confirmed patients with COVID-19. Second, the “Psychometric Evaluation of Healthcare Workers’ Stress Related to Caring for Patients with a Highly Infectious Disease” scale developed by Chuang and Lou for 2003 SARS was used.^7^ The stress scales were divided into four subscales, which measured the worry of social isolation (10 items), discomfort caused by the protective equipment (8 items), difficulties and anxiety regarding infection control (7 items) and the workload of caring for patients (7 items). Each of these 32 items was rated on a four-point Likert scale (0: not at all, 1: about the same as usual, 2: slightly more severe than usual, 3: more severe than usual) to assess the degree of stress caused by various factors. The total score could range from 0 to 96. A higher total score would indicate a greater degree of stress, such that a total score from 47 to 96 would denote severe stress, while a score from 0 to 46 would indicate mild to moderate stress. A score of 0 would indicate absence of stress.

### Statistical analysis

The software package JMP 13.0 for Windows (SAS Institute Inc, Cary, North Carolina, United States) was used for statistical analysis. Continuous variables relating to demographic characteristics and perceived work stress were presented as the mean ± standard deviation (SD); categorical variables were presented as counts and percentages. Correlations between variables were analyzed using Pearson’s product-moment correlation coefficient and were compared using the chi-square and Levene tests. Logistic regression on different variables was also used to evaluate the effect of the variables on the total stress scale.

### Ethics

This study was reviewed and approved (IRB Number: KMUHIRB-E(I)-2020008; approval date: April 7, 2020) by the institutional review board of the participating hospital. Data were collected anonymously, and background data were deidentified. Information about the respondents that was obtained in this study was handle in accordance with the principles of confidentiality and privacy.

## RESULTS

### Characteristics of participants

Out of the 492 healthcare workers who completed this questionnaire, 51 were male (10.4%) and 441 were female (89.6%). The demographic characteristics of these healthcare workers are displayed in Table 1.

**Table 1.** Demographic characteristics of the healthcare workers (n = 492)

Characteristic	Healthcare workers
Median age (IQR), years	38 (23-65)
Gender, n (%)	
Male	51 (10.4)
Female	441 (89.6)
Work experience, mean (SD), years	12.4 (9)
Marital status, n (%)	
Unmarried	243 (49.4)
Married	249 (50.6)
Number of children, n (%)	
≥ 1	215 (43.7)
none	277 (56.3)
Occupation, n (%)	
Doctor	40 (8.1)
Nurse	404 (82.1)
Medical technician	48 (9.8)
Educational level, n (%)	
College/university	427 (86.8)
Institute (MSc and PhD)	65 (13.2)
Experience with SARS, n (%)	
No	435 (88.4)
Yes	57 (11.6)
Caring for patients with suspected or confirmed COVID-19, n (%)	
No	399 (81.1)
Yes	93 (18.9)

IQR = interquartile range; SD = standard deviation; SARS = severe acute respiratory syndrome.

Their mean age was 38 years (ranging from 23 to 65 years) and their average length of work experience was 12.4 years. Approximately half of the respondents were married (50.6%) and were parents (43.7%). Most of the respondents were nurses (82.8%), followed by medical technicians (9.8%) and doctors (8.1%). A total of 427 respondents (86.8%) had graduated from college or university at not more than bachelor level, while the remaining respondents (13.2%) had also reached higher degree levels (master’s or doctoral degree). Ninety-three respondents (18.9%) had prior work experience at the time of the SARS outbreak and 57 (11.6%) were frontline healthcare workers who were directly involved in the diagnosis, treatment and care of patients with COVID-19.

### The stress levels of healthcare workers amidst the COVID-19 pandemic

The total stress score was collated and analyzed in relation to different variables (see Table 2). The respondents aged 41 years or above (score of 50.9 ± 16.4) reported having significantly higher stress scores than those below 40 years of age (46.4 ± 16.0), when facing the COVID-19 pandemic (P = 0.0026). In addition, female healthcare workers reported significantly higher stress levels than males (49.0 ± 16.5 versus 43.5 ± 13.7; P = 0.0109). Respondents who were married (50.1 ± 16.9) reported higher levels of stress than those who were unmarried (46.7 ± 15.5) (P = 0.0186) and respondents who were parents (50.1 ± 17.3) reported significantly higher stress scores than those who had no child (P = 0.0409). Furthermore, nurses reported the highest total stress score (49.5 ± 16.2; P = 0.0063), followed by medical technicians (44.6 ± 14.8) and doctors (42.3 ± 17.7). The following factors: educational level, prior work experience with SARS and whether having cared for patients with suspected or confirmed COVID-19 infection, had no significant influence on the total stress score. Total stress scores from 47 to 96 indicate severe stress and scores from 0 to 46 indicate mild to moderate stress.

**Table 2.** Total stress scores among the healthcare workers facing the COVID-19 pandemic

Variable	Number (%)	Total stress scale (mean ± SD)	T	P-value
Age (23 to 65 years)				
≤ 40	263 (53.5)	46.4 ± 16.0	−3.03	0.0026*
> 41	229 (46.5)	50.9 ± 16.4		
Gender				
Male	51 (10.4)	43.5 ± 13.7	2.62	0.0109*
Female	441 (89.6)	49.0 ± 16.5		
Marital status				
Unmarried	243 (49.4)	46.7 ± 15.5	2.36	0.0186*
Married	249 (50.6)	50.1 ± 16.9		
Number of children				
≥ 1	215 (43.7)	50.1 ± 17.3	2.05	0.0409*
None	277 (56.3)	47.1 ± 15.4		
Occupation				
Doctor	40 (8.1)	42.3 ± 17.7	F:5.12	0.0063* (2 > 1)
Nurse	404 (82.1)	49.5 ± 16.2		
Medical technician	48 (9.8)	44.6 ± 14.8		
Educational level				
College/university	427 (86.8)	48.8 ± 15.8	1.15	0.2539
Institute (MSc and PhD)	65 (13.2)	45.9 ± 19.3		
Experience of SARS				
No	435 (88.4)	47.8 ± 15.7	1.95	0.0560
Yes	57 (11.6)	53.2 ± 20.0		
Caring for patients with suspected or confirmed COVID-19 infection				
No	399 (81.1)	51.4 ± 17.3	1.94	0.0529
Yes	93 (18.9)	47.8 ± 16.0		

*P < 0.05; SD = standard deviation; SARS = severe acute respiratory syndrome.

Further analysis via logistic regression on different variables (Table 3) showed that the odds of being in the group with severe stress increased significantly when respondents were older (adjusted odds ratio, OR: 1.028; P = 0.028) or nurses (compared with medical technicians, adjusted OR: 2.075; P = 0.037). Although not statistically significant, respondents who were married (adjusted OR: 1.736; P = 0.138) or doctors (compared with medical technicians, adjusted OR: 1.222; P = 0.669) showed a positive relationship with being in the group with severe stress. On the contrary, those who were male (adjusted OR: 0.659; P = 0.296) or parents (adjusted OR: 0.720; P = 0.385) showed a negative relationship with being in the group with severe stress.

**Table 3.** Factors affecting the odds of being in the group with severe stress

Variables	Crude OR (95% CI)	P-value	Adjusted OR (95% CI)	P-value
Age	1.033 (1.013-1.053)	0.001*	1.028 (1.003-1.054)	0.028*
Gender (male: female)	0.497 (0.269-0.896)	0.022*	0.659 (0.296-1.432)	0.296
Marital status (married: unmarried)	1.686 (1.181-2.413)	0.004*	1.736 (0.844-3.663)	0.138
Number of children (≥ 1: none)	1.533 (1.071-2.199)	0.020*	0.720 (0.337-1.494)	0.385
Occupation				
Doctor Nurse Medical technician	1.232 (0.522-2.917) 2.074 (1.130-3.906) Reference	0.633 0.020*	1.222 (0.488-3.083) 2.075 (1.052-4.181) Reference	0.669 0.037*
Experience of SARS				
SARS (+)^c^ SARS (-)^d^	1.936 (1.095-3.530) Reference	0.026*	1.516 (0.827-2.858) Reference	0.186

OR = odds ratio; CI = confidence interval; SARS = severe acute respiratory syndrome; SARS (-): no experience of SARS; SARS (+): experience of SARS; . *P < 0.05.

### Influence of SARS work experience on healthcare workers

The healthcare workers were divided into two groups according to whether they had experience of treating SARS. The total stress score and the scores from the four subscales were analyzed in each group (see Table 4). During the COVID-19 pandemic, although not statistically significant, the healthcare workers with SARS work experience had higher total stress levels (score of 53.2 ± 20.0) than those with no SARS work experience (47.8 ± 15.7). Further analysis on the four subscales revealed that the healthcare workers with experience of treating SARS reported having significantly higher stress scores on two of the four subscales, i.e. discomfort caused by the protective equipment and workload of caring for patients (16.5 ± 5.4 versus 14.8 ± 4.9; 13.2 ± 4.9 versus 11.1 ± 4.5, respectively), amidst the COVID-19 outbreak. On the other hand, there was no significant difference in subscale scores relating to the worry of social isolation and the difficulties and anxiety regarding infection control, among the healthcare workers during the COVID-19 outbreak, between those with and without experience of treating SARS. For 57 respondents with SARS work experience, 38 respondents (66.7%) were in the group with severe stress and the remaining 19 respondents (33.3%) were in the group with mild to moderate stress. Further analysis via multivariable logistic regression (Table 3) showed that, compared with the healthcare workers with no SARS work experience, those with SARS work experience (adjusted OR: 1.516; P = 0.186) had a positive relationship with being in the group with severe stress.

**Table 4.** Psychometric evaluation on the healthcare workers with or without experience of severe acute respiratory syndrome (SARS) amidst the COVID-19 pandemic

Subscale	All n = 492	SARS experience		T/x^2^	P-value
		Yes, n = 57	No, n = 435		
Worry of social isolation	13.0 ± 6.3	14.7 ± 7.8	12.8 ± 6.0	1.77	0.0817
Discomfort caused by the protective equipment	15.0 ± 5.0	16.5 ± 5.4	14.8 ± 4.9	2.36	0.0185*
Difficulties and anxiety regarding infection control	9.0 ± 3.8	8.7 ± 4.9	9.0 ± 3.7	0.43	0.6705
Workload of caring for patients	11.4 ± 4.6	13.2 ± 4.9	11.1 ± 4.5	3.25	0.0012*
Total stress scale	48.4 ± 16.3	53.2 ± 20.0	47.8 ± 15.7	1.95	0.0560
Mild to moderate stress^a^, number (%)	233 (47.4)	19 (33.3)	214 (49.2)	5.09	0.0241*
Severe stress^b^, number (%)	259 (52.6)	38 (66.7)	221 (50.8)		

^a^Mild to moderate stress: total score 0-46; ^b^Severe stress: total score 47-96; *P < 0.05.

### Stress scores of healthcare workers according to two factors

The respondents were categorized into four groups according to two factors, namely, SARS work experience and caring for suspected or confirmed COVID-19 cases (refer to Table 5). The frontline healthcare workers with SARS experience had the highest total stress score (56.4 ± 20.2), followed by non-frontline healthcare workers with SARS work experience (51.6 ± 19.9), frontline healthcare workers with no SARS work experience (50.1 ± 16.4), and non-frontline healthcare workers with no SARS work experience (47.3 ± 15.5). Two of the four subscales (the worry of social isolation and the workload of caring for patients) showed increasing trends regarding the total stress score among the healthcare workers. Furthermore, the frontline healthcare workers with SARS work experience (14.4 ± 4.5) demonstrated significantly higher stress levels than the non-frontline healthcare workers with no SARS work experience (10.9 ± 4.5), in relation to the subscale of the workload of caring for patients.

**Table 5.** Psychometric evaluation on the healthcare workers, in four subgroups according to their experience of severe acute respiratory syndrome (SARS) and whether they were caring for suspected or confirmed COVID-19 cases

Subscale	SARS (−)		SARS (+)		F	P value	Tukey’s HSD
	1. COVID (−) n = 38	2. COVID (+) n = 19	3. COVID (−) n = 361	4. COVID (+) n = 74			
Worry of social isolation	12.6 ± 6.1	13.8 ± 5.7	14.0 ± 7.7	16.2 ± 8.0	2.73	0.0434	No difference
Discomfort caused by the protective equipment	14.8 ± 5.0	15.0 ± 5.0	16.3 ± 5.8	16.8 ± 4.6	1.92	0.1250	
Difficulties and anxiety regarding infection control	9.0 ± 3.5	9.2 ± 4.3	8.6 ± 4.7	8.9 ± 5.3	0.21	0.8915	
Workload of caring for patients	10.9 ± 4.5	11.2 ± 4.6	12.7 ± 5.0	14.4 ± 4.5	5.71	0.0080	4 > 1*
Total stress scale	47.3 ± 15.5	48.1 ± 16.3	51.6 ± 19.9	56.4 ± 20.2	2.79	0.0402	No difference

SARS (-) = no experience of SARS; SARS (+) = experience of SARS; COVID (-) = non-frontline, not caring for patients with suspected or confirmed COVID-19 infection; COVID (+) = frontline, caring for patients with suspected or confirmed COVID-19 infection; Tukey’s HSD = Tukey’s Honestly Significant Difference test; *P < 0.05.

## DISCUSSION

The cross-sectional survey used in this study was completed by 492 respondents and the mean total stress score was 48.4 ± 16.3 points. Most participants were female, were nurses, had been educated to college or university level and had no prior experience of treating SARS.

In this survey, the total stress score was significantly higher among healthcare workers aged 41 or above who were female, married, parents and nurses. The total stress score did not show any significant differences with regard to education level, having SARS work experience or having cared for suspected or confirmed cases of COVID-19. Furthermore, respondents who were older or were nurses had higher odds of being in the group with severe stress during the COVID-19 outbreak.

Nurses not only had higher total stress scores than physicians or medical technicians, but also had significantly higher odds of being in the group with severe stress in facing the COVID-19 pandemic. This was consistent with previous findings that demonstrated that SARS had caused a significant level of distress among emergency department staff, with the highest levels of distress among nurses, followed by doctors and healthcare assistants.^8^ The higher stress among nurses was attributable to the following reasons: nursing is a highly stressful occupation; nurses are exposed to patients with suspected or confirmed COVID-19 for long periods; and the inconvenience caused by stringent infection control measures is a source of stress.

Being a parent also gave rise to a higher total stress score in this study. This may be attributed to the fear among these healthcare workers that if they contracted COVID-19, they could spread the disease to their children, be separated from and not able to see their children, or would face inconvenience in taking care of their children. Furthermore, a recent study conducted in tertiary-level hospitals found that nurses, women, frontline workers and workers at the epicenter of COVID-19 reported experiencing greater severity of symptoms of depression, anxiety, insomnia and distress.^9^

One intriguing finding from the present study was that healthcare workers with SARS work experience presented significantly higher stress scores on the subscales relating to discomfort caused by the protective equipment and the workload of caring for patients. However, no significant differences in the total stress score were found.

Prior experience of treating SARS had either positive, negative or neutral psychological impacts on the healthcare workers when they were faced with another pandemic; in this case, COVID-19. A survey completed by healthcare workers who practiced respiratory medicine during the SARS outbreak in Hong Kong showed that these workers remained highly stressed one year after the outbreak. The perceived stress levels were higher and were associated with higher levels of depression and anxiety, and higher posttraumatic stress scores.^10^ A study on healthcare workers in Taiwan who had taken care of suspected SARS patients, both at the first stage and a year later, indicated that the stress was initially in response to the life-threatening nature of the SARS epidemic. The stress experienced thereafter was from their jobs, families and stressful events within daily life, rather than from a continuation of previous symptoms caused by vulnerability to or complications from stress created by the SARS attack.^11^ In another study, SARS work experience resulted in increased mental preparedness and implementation of stringent infection control measures that led to lower impact of events on stress scores among physicians and nurses, as well as lower prevalence rates for posttraumatic stress disorder in response to the COVID-19 pandemic.^12^

A complete set of personal protective equipment (PEE) is imperative and paramount for curbing the spread of COVID-19 when dealing with suspected or confirmed cases. However, healthcare workers who wear a complete set of personal protective equipment have experienced states of tension and fatigue, thereby increasing the difficulty of their work and becoming more prone to burnout.^13^ In the present study, the healthcare workers with SARS work experience had higher stress scores due to the discomfort experienced through using personal protective equipment. The three most troublesome effects were the following: skin damage to their hands from frequent hand washing and use of disinfectants; the inconvenience of going to the bathroom wearing PPE; and restriction of the intake of water and food because of wearing a complete set of PPE.

Because hand hygiene compliance is essential amidst the COVID-19 pandemic, prevention of skin dermatitis caused by constant hand washing and sanitizing among healthcare workers has been found to be important for increasing hand hygiene compliance.^14^ For lipophilic enveloped viruses, such as SARS-CoV-2, it has been emphasized that alcohol-based sanitizers should have a good quantity of antimicrobial properties and should have good skin tolerability, compared with handwashing with soap and water. This would reduce the potential for skin dermatitis.^15^

In this study, we categorized participants as those who had prior experience of treating SARS and those without prior SARS work experience. These two groups were further divided into four subgroups depending on whether they were or were not caring for suspected or confirmed cases of COVID-19. There was an upward trend in the total stress scores among healthcare workers who had SARS work experience and were caring for suspected or confirmed cases of COVID-19. The highest stress scores were seen among frontline healthcare workers with SARS work experience. Two subscales, the worry of social isolation and the workload of caring for patients, revealed similar increasing trends in stress scores. Furthermore, compared with non-frontline healthcare workers with no SARS work experience, our analysis indicated that there were significantly higher stress scores among frontline healthcare workers with SARS work experience, in relation to the subscale of the workload of caring for patients. Consistent with previous findings, frontline healthcare workers were found in our study to have experienced the highest psychological burden during the COVID-19 pandemic.^9^ Moreover, our study implied that prior experience of treating SARS might be a factor contributing negatively to the stress levels experienced by frontline healthcare workers during the COVID-19 outbreak.

The importance and benefits of psychiatric measures or interventions for high-risk healthcare workers amidst pandemics, towards reducing their stress levels, have been emphasized in several studies. It was demonstrated that psychiatric services were significantly effective in helping healthcare workers manage their stress during the SARS outbreak.^16^ During the COVID-19 pandemic, a psychological intervention plan was proposed, which included measures within three main areas: first, construction of a medical team for psychological interventions, with provision of online courses to guide medical staff in dealing with common psychological problems; second, launching of a psychological assistance hotline team offering guidance and supervision for solving psychological problems; and third, implementation of these psychological interventions.^17^ The psychological intervention measures should be based on the needs of healthcare workers and should be tailored to different cultural backgrounds, religious beliefs and personal preferences.

The present study has several limitations. First, it was limited in scope because it only investigated a limited number of healthcare workers in one tertiary-level hospital in southern Taiwan. Thus, the results cannot be generalized to all Taiwanese healthcare workers. Likewise, this study did not include nonmedical personnel in the hospital (e.g. allied healthcare professionals, pharmacists, administrators, clerical staff and maintenance workers) who may also have suffered from psychological distress amidst the COVID-19 outbreak. Second, because of the cross-sectional design of this study, it was only able to assess healthcare workers’ work stress at the time of the survey. It thus lacked longitudinal observation of the participants. Third, apart from the factors identified in this study, there may be additional factors contributing to work stress among healthcare workers. Fourth, the non-inclusion of a scale for evaluating depression was also a limitation of this study.

## CONCLUSIONS

In summary, healthcare workers who are older than 40, female, married, parents or nurses are prone to have higher total stress scores amidst the COVID-19 pandemic. The odds of being in the group with severe stress were significantly higher when the respondents were older or nurses. Furthermore, healthcare workers with SARS work experience reported having higher total stress scores and had higher odds of being in the group with severe stress, although this was not statistically significant compared with healthcare workers without SARS work experience. Specifically, healthcare workers with experience of treating SARS showed greater distress on the subscales relating to the discomfort caused by the protective equipment and the workload of caring for patients in the COVID-19 pandemic. In addition, frontline healthcare workers with SARS work experience also had higher stress scores on the subscale of the workload of caring for patients.

Due to limitations of workforce, finance and time, data for this study were collected only from one medical center in southern Taiwan. It is recommended that future studies should include larger sample sizes and should make comparisons between groups with the same numbers of study subjects in other tertiary-level hospitals in Taiwan. Moreover, a longitudinal study design should be adopted in order to follow up the long-term effect of COVID-19 on healthcare workers.

It was clear from this study that experience of treating the 2003 SARS outbreak had a partly negative psychological impact on these healthcare workers facing the COVID-19 outbreak. Thus, hospital administrators should provide psychological measures or interventions in order to reduce the burden on healthcare workers amidst the COVID-19 pandemic.
